# CGHregions: Dimension Reduction for Array CGH Data with Minimal Information Loss

**Published:** 2007-02-08

**Authors:** Mark A. van de Wiel, Wessel N. van Wieringen

**Affiliations:** 1 Department of Pathology and Department of Biostatistics (KEB), VU University Medical Center, Amsterdam, The Netherlands; 2 Department of Mathematics, Vrije Universiteit, Amsterdam, The Netherlands

**Keywords:** Array CGH, Dimension reduction, Tumor profiles, Statistical testing, FDR

## Abstract

An algorithm to reduce multi-sample array CGH data from thousands of clones to tens or hundreds of clone regions is introduced. This reduction of the data is performed such that little information is lost, which is possible due to the high dependencies between neighboring clones. The algorithm is explained using a small example. The potential beneficial effects of the algorithm for downstream analysis are illustrated by re-analysis of previously published colorectal cancer data. Using multiple testing corrections suitable for these data, we provide statistical evidence for genomic differences on several clone regions between MSI+ and CIN+ tumors. The algorithm, named CGHregions, is available as an easy-to-use script in R.

## Introduction

Array Comparative Genomic Hybridization (array CGH) is an increasingly popular high-resolution technique to discover at which chromosomal locations the DNA is aberrated. We refer to such a location by the array element covering it, a ‘clone,’ which technically could also be a synthetic oligonucleotide.

An aberration may be a ‘loss’ of at least one chromosomal copy or a ‘gain’ of at least one copy. In some cases, one distinguishes one extra state: ‘amplification’ which is a multiple copy number gain. Non-aberrated clones are referred to as ‘normal.’ Among others, cancer is an important application area of this technique. It has been successfully applied to discover genomic disorders in tumors. Also, due to the stability of the DNA molecule (as opposed to mRNA) it has potential to be used in a practical diagnostic context (Pinkel and Albertson, 2005). Integration of these data with mRNA gene expression data is currently actively studied (van Wieringen et al. 2006a), since these two data types measure the activity of genes at different levels: genome and transcriptome.

As with gene expression, array CGH data requires pre-processing and normalisation. The nature of the data is such that sudden state changes, which we refer to as ‘transitions’, occur along the DNA. Moreover, the measurements, which are log2-ratios with respect to a normal ‘two-copy everywhere’ sample, reflect hidden underlying discrete states (loss, normal, gain). Therefore, before starting with downstream analysis, one usually divides the measurements into ‘segments’ with similar log2-ratios and discretizes the log2-ratios back to these three states. These two processes are termed segmentation and calling. Many have proposed methods to automate this process. We refer to Willenbrock and Fridlyand (2005) and Lai et al. (2005) for comparisons of several methods. After calling, the data, which is ordered along the DNA, may be represented as sequences containing −1, 0, 1, which code for loss, normal and gain.

For further downstream analysis (testing, clustering, classification) the starting point would be a large matrix of, say, *C* rows (clones) and *N* columns (samples) containing these codes. Our goal is to reduce the size of this matrix significantly by determining sequences of clones which for every sample are (almost) constant within the sample. Hence, it is a multi-sample approach and sequences are shared by all samples. Naturally, transitions occur at different locations for different samples, but we observed that, in general, many samples representing the same type of tissue have a large overlap in sequences of clones that do not change. We denote such a sequence of clones as a region. Note that we do not require the clones in a region to be constant *across* samples. We show that this dimension reduction is feasible with very limited and controlled information loss.

The benefits of such a dimension reduction are several. Obviously, decreasing *C* with a factor of 10 to 250 aids in reducing computing times for downstream analyses, which may be useful especially when *N* is large. More importantly, it eases interpretation for biologists who may study the limited number of DNA-regions rather than a huge collection of clones. Moreover, it has a positive impact on the effectiveness of downstream analysis. The latter is illustrated for colorectal cancer data (Douglas et al. 2004) in a two-sample testing situation. We show that the multiple testing problem is reduced by combining our region construction algorithm with a dedicated False Discovery Rate (FDR) correction (Gilbert, 2005). While the clone-wise testing did not result in any statistically significant differences after FDR correction, several clone regions were found to be significant. This enabled us to provide statistical backup for findings previously published by Douglas et al. (2004).

## Methods

### Construction of regions

The input for our method is a *C* × *N* matrix *A* of which entry *a_is_* = *r* represents the level for clone *i* and sample *s*, where *r* = −1, 0, 1 (loss, normal or gain). A row of this matrix is called the (clone) aberration signature, while a column is the (sample) profile. A region is simply a collection of subsequent rows of *A*. At each stage of the algorithm a region is identified by an index *k* which denotes the order in which regions appear in *A*. Then, a region *k* is a sequence of *l_k_* clones represented by *A_k_* = (*A_k,_* _1_, …, *A_k,l_k__*), where *A*_*k,*1_ denotes the *i*th data row for region *k*.

The regions are constructed by restricting the maximum distance between any two clones in a region by threshold *c*. First, we assume *c* to be fixed; determining its value is discussed later. A final region *k* is a sequence of clones on the chromosomes that satisfies the following condition:

(1) $$d(A_{k,i},A_{k,j})\leq c,\;\;\text{for}\;\;\text{all}\;i,j,$$

where *d*( ) is the *L*_1_-distance function. For example, *d* ((0, 0, 1, −1, 0), (0, 1, 1, 1, −1)) = |0| + |−1| + |0| + |−2| + |1| = 4. Hence, it is basically counting how often two signatures disagree, where a loss-gain disagreement is double-counted. We require that this condition is always satisfied. It does not uniquely define the region boundaries. We apply three breakdown rules to define the boundaries. First, recognizing the physical boundaries between chromosomes, a break is always inserted before the first clone of a chromosome. This introduces a limited number of preliminary regions *k* = 1, ... , *K* each denoted by ${A^{\prime }}_{k}=({A^{\prime }}_{k,1},\ldots ,{A^{\prime }}_{k,l_{k}})$, where *l_k_* is the number of clones in region *k*. Second, the nature of array CGH data is such that signature changes are likely to occur suddenly at certain positions. Therefore, a break is inserted before position *A′_k, i_* if

(2) $$d({A^{\prime }}_{k,i-1},{A^{\prime }}_{k,i})>c.$$

After application of (2) the majority of regions usually satisfies (1). However, some regions may not satisfy (2) because of more gradual changes. In order to satisfy (1) we insert a break before or after the clone for which the ‘gradient’ is largest. For this discrete setting, we define the gradient for clone *i* in region *k* as

(3) $$g({A^{\prime }}_{k,i})=\frac{\sum_{j=1}^{l_{k}}w_{ij}d({A^{\prime }}_{k,i},{A^{\prime }}_{k,j})}{\sum_{j=1}^{l_{k}}w_{ij}},$$

where *w_ij_* = 0 if *i* = *j* and *w_ij_* = 1/|*j* – *i*|, otherwise. To decide whether to insert the break before or after the clone with maximum gradient, we compute the left- and right-gradient, *g_l_* and *g_r_* with similar definitions as *g*. However, only clones to the left or right contribute to *g_l_* and *g_r_*, respectively. Then, we insert the break before the *i*th clone if $g_{l}({A^{\prime }}_{i})\geq g_{r}({A^{\prime }}_{i})$ and after, otherwise.

The gradient rule is iteratively applied until condition (1) is satisfied. The entire algorithm is symmetric, which means that the same regions are formed when starting the break insertion from the left or the right.

### Robustification of the algorithm

The algorithm may pick up so-called mono-regions, which are regions consisting of one clone only. While in principle such a region could be real, it is very likely that the clone is wrongly positioned. This happens regularly due to mis-mapping of the genomic sequence to the position of the clone on the DNA. Therefore, such a clone may appear to be an outlier between its ‘neighbors’. We prefer to have an algorithm which is robust against such mis-mapping and have therefore included the (recommended) option to delete such mono-regions.

When mono-regions are deleted, the algorithm needs an extra step to decide whether adjacent regions need to be concatenated. If (2) is satisfied for the two clones adjacent to the deleted mono-region, a break is inserted and the rest of the two adjacent regions remains unchanged. If not, the two adjacent regions are connected and condition (1) is checked. If it is satisfied, we keep the new region, otherwise the gradient rule is iteratively applied to break the region in pieces until (1) is satisfied.

### Representative signature

Once the regions are defined, we have to choose a representative signature, after which the data for a region will collapse into the boundaries of the region and one signature only. We propose the medoid of all signatures in the region as the representative signature, which is that signature with least average distance to the other signatures. Conventional computation of the medoid would require quadratic time, because for each clone the distances to all the other clones need to be computed. We developed a more efficient computation of this medoid, which we discuss later.

### Example

We illustrate the algorithm for a toy example with 10 samples and 18 clones for 2 chromosomes. The data are displayed in Table 1, where −1, 0, 1 indicate loss, normal and gain, respectively.

We illustrate the algorithm for *c* = 2. First, a break is inserted after clone 3, since clone 4 lies on the next chromosome. Then, we apply (2) and breaks are inserted before clones 1, 7, 8, 10 and 17. Then we have six regions: 1–3, 4–6, 7, 8–9, 10–16 and 17–18. These all satisfy basic condition (1), except for the fifth region, because the distance between its first and 5th clone exceeds *c* = 2: $d({A^{\prime }}_{5,1},{A^{\prime }}_{5,5})=3$. The maximum gradient as computed from (3) is attained at its fourth clone (clone 13): $g({A^{\prime }}_{5,4})$ = (2/3 + 2/2 + 2/1 + 1/1 + 1/2 + 1/3)/(1/3 + 1/2 + 1 +1 + 1/2 + 1/3) = (11/2)/(11/3) = 3/2. Its left-gradient is larger than its right-gradient, so a break is inserted before this clone. The two regions created satisfy (1). Finally, we delete the mono-clone, clone 7 and concatenate regions 4–6 and 8–9 to form one region, which satisfies (1). Then we have the following regions: 1–3, (4, 5, 6, 8, 9), 10–12, 13–16 and 17–18. We compute the medoids for these regions and reduce the data to those displayed in Table 2.

### Choice of *c*

For imperfect data, wrong calls of aberrations may cause nonsense and random transitions in each sample. Even for perfect data one may argue that transitions that occur locally for only a small number of individuals may not be relevant. However, if such transitions are of a more global (across genome) character consistently for a small number of individuals, one may want to pick these up. The automatic choice of threshold *c* is such that only very little information is lost when representing the regions by their medoid signatures. In short, we set threshold *c* to *c*_max_, with

(4) $$c_{\text{max}}=\text{arg}\;\;\text{max}\{c\in \mathbb{N}:a(c)\leq T\},$$

where *a*(*c*) is a measure of prediction error when one replaces the individual clone data by the medoid signature of the region it belongs to. Roughly, *T* is the maximally sustained proportion of mis-predictions. Denoting the medoid signature of region *k* by $A_{k}^{m}$, we define *a*(*c*) as

(5) $$\begin{array}{l} a(c)=\left(\sum_{k\in \text{K}}\sum_{i=1}^{l_{k}}a_{k,i}\right)/L\;with\\ a_{k,i}(c)=d\left(A_{k,i},A_{k}^{m}\right)/N, \end{array}$$

where **K** is the set of clone regions with relatively many aberrations in $A_{k}^{m}$ (default: top 25%) and *L* is the total number of clones in these ‘active’ regions. We restrict the computations to active regions, since array CGH data may contain large portions of normals which are less interesting to the biologist. Hence, it is preferred to have little information loss in those ‘active’ regions. Note that the exclusion of mono-clones does not influence *a*(*c*), because $d(A_{k,i},A_{k}^{m})=0$ for such clones. Moreover, for *c* = 0 (forcing a break at every change), we have no information loss, *a*(0) = 0.

Using (4) we quantify how much information is maintained when summarizing the clones and their signatures by the region boundaries and their representative signatures. Formulating (4) in terms of proportions rather than absolute numbers avoids sample size bias: the number of regions depends on the number of transitions that are persistent over a reasonably large proportion of the samples. If there exists a small subset of samples (say, for example, 10%) with many transitions located elsewhere than for the 90% majority, a too large *c* may not distinguish regions from adjacent regions when their signatures differ only for the 10% minority of the samples. Then, those regions would be merged. However, by using (4) one avoids this: if *c* is chosen too large, the region boundaries will be mostly determined from the 90% majority. Therefore, many regions contribute to *a* (*c*), because the 10% subset has transition locations within those regions and (4) will be violated. In general, if the tumor sample group is heterogenous (transitions occur at different places), *c*_max_ is smaller than in the case of a homogeneous group and hence more regions will be created for sets of samples with heterogenous transition location patterns.

In (4), *T* can be interpreted as the maximal information loss that is allowed. Increasing *T* decreases the number of regions. In general, we recommend to use *T* = 0.01, so that very little information is lost. However, in case one aims to perform statistical tests on small subgroups of tumor samples (say at least one group containing less than 10 samples), we recommend to use *T* = 0.025 to possibly increase statistical power due to less severe multiple testing corrections.

The reduction effect of the algorithm depends on the platform (e.g. BAC or oligonucleotide), the heterogeneity of the transition locations among the tumor samples and the value of *T*. For BAC array data with approximately 4000 clones, we observed reduction factors of 10 to 30 using *T* = 0.01 and 20 to 50 using *T* = 0.025. For oligonucleotide data with approximately 24000 oligos, those reduction factors were 40 to 100 and 60 to 250 for *T* = 0.01 and *T* = 0.025, respectively.

### Example, continued

Suppose we accept maximally 1% information loss, so we set *T* = 0.01. We assume that the regions displayed in Table 2 are the 25% ‘active’ ones in a larger set, hence forming **K** in (5). From Tables 1 and 2 we observe that only one clone, clone 13, deviates from its representative signature, and it does so at one position. Hence, *a*(2) = (1/10)/17 = 0.0059, so the inequality in (4) is satisfied for *c* = 2. Now, set *c* = 3. Then, clones 10–12 and 13–16 form one region, 10–16 and the rest remains unchanged with respect to *c* = 2. Region 10–16 has the signature of clone 13 as its medoid. Then, *a*(3) = ((1 + 3 * 2 + 3 *1)/10)/17 = 0.059 > 0.01, so the inequality in (4) is violated for *c* = 3. Therefore, *c* = *c*_max_ = 2 is chosen.

### Computational issues

We increased computational efficiency of CGHregions in two ways. Firstly, *c* is tuned to satisfy (4) using only a fraction of the clones of the original data set. This subset of clones consists of three blocks of 400 subsequent clones. The value of *c*_max_ found for this smaller data set serves as an initial value of *c* on the entire data set. Secondly, we implemented a computation of the medoid tailored to this discrete data. For each sample, the number of level *r* observations (*r* = −1, 0, 1) within the region under study, say region *k*, is counted. We obtain a *N* × 3 matrix *M* of which element *m_sr_* represents the count for sample *s* and level *r*. The total distance of the clone *i* signature *A_k, i_* = (*A_k, i,_* _1_, ..., *A_k, i, N_*) to the rest of the signatures in the region is then obtained by multiplying the distance between *A_k, i, s_* and *r* by the count of *r* in sample *j* (i.e. *m_sr_*) and summing the results over *r* = −1, 0, 1 and over *s* = 1, ..., *N*. Then, the medoid for region *k* is that signature *i* with minimal total (and hence also average) distance. This computation is linear in the number of clones in region *k*, while conventional computation of total distances for all clones would be quadratic in these number. These computational adjustments reduced computing time by several factors allowing the construction of regions within minutes for most data sets.

### Implementation

CGHregions is implemented using the statistical software environment R (R Development Core Team (2006)). The scripts and example data are available from the author’s web site: http://www.few.vu.nl/~mavdwiel/CGHregions.html. The scripts contain instructions for running the algorithm, which requires almost no knowledge of R.

## Results: Application to Colorectal Cancer Data

We show the positive effect of using regions rather than individual clones in a two group testing context. Douglas et al. (2004) discuss an array CGH data set containing cell line and colorectal tumor samples, respectively. Here, we focus on partly re-analyzing the tumor data. These consist of two groups: 7 microsatellite instable (MSI+) and 30 chromosomal instable (CIN+) samples. Among many other questions, the authors are interested in genomic differences between the two groups and the chromosomal locations of these differences. When comparing the proportions of aberrations between the MSI+ and CIN+ group, Douglas et al. (2004) (see p. 4820) report mainly about entire chromosomal arms (8p, 17p, 18q) or chromosomes (20), although exceptions are made for one individual clone and a small region on 18q. The authors do not mention statistical measures (such as *p*-values) of the differences reported, except for the difference in proportion of gain on chromosome 20. This *p*-value, however, is not corrected for multiple testing, which is recommended for microarray studies (Allison et al. 2006). Multiple testing correction is a real challenge for these data: the discrete levels and the small sample size of the MSI+ group prevent very small *p*-values. Hence, False Discovery Rates (FDR) or Family Wise Error Rates (FWER) tend to be large for such data. We demonstrate that reducing the clones to clone regions aids in solving this problem.

**Figure 1.**
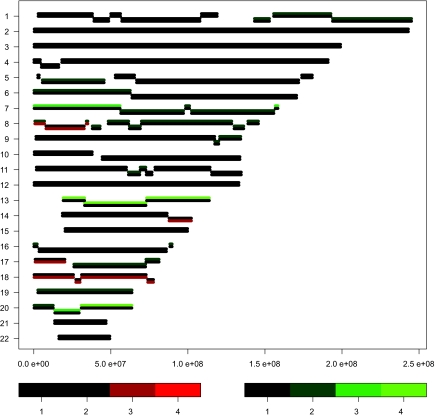
Visualization of 68 regions created by CGHregions for 37 colorectal tumor samples (Douglas et al. 2004). Y-axis: chromosome, X-axis: base pair position. A new region is displayed by a slight jump with respect to the previous region. The number of regions per chromosome ranges from 1 (several) to 9 (chromosome 8), indicating that the resolution of the results adapts to the heterogeneity of the transition locations. Each region is displayed as a bi-colored segment, the lower and upper part of which correspond to the proportions p_l_ and p_g_ of samples with a loss (red) or gain (green), respectively. The color coding is displayed as well: ‘1’: *p_l_* (*p_g_*) < 10%; ‘2’: 10% ≤ *p_l_* (*p_g_*) < 30%; ‘3’: 30% ≤ *p_l_* (*p_g_*) < 50%; ‘4’: *p_l_* (*p_g_*) ≥ 50%.

The data, available at the supplementary website of Douglas et al. (2004), were first filtered (clones with less than 30% missings were kept), segmented using DNAcopy (Olshen et al. 2004) and then segment-wise aberrations were called using a Normal mixture model. From the resulting clone data set, the clone region data set was produced using the above algorithm with *T* = 0.025. Since we have 37 samples, this threshold roughly means that one allows on average one clone per 37 to deviate from the region-wise signature. The number of items was reduced from 3129 autosomal clones to 68 clone regions (Figure 1). To both data sets (clone-wise calls and region-wise calls) we applied a Wilcoxon two-sample test corrected for ties (van de Wiel et al. 2005) to compute exact *p*-values for the difference in levels between the two groups. Finally, a modified Benjamini-Hochberg FDR correction was applied to the *p*-values. The modification was proposed by Gilbert (2005) to render more effective FDR-control for discrete level data.

For the clone data set, the smallest uncorrected *p*-value equals 0.001202 which corresponds to an FDR-value of 0.3815. This value is reached for 5 subsequent clones on chromosome 8. Table 3 displays the most significant results for the region data set. It is obvious that for the single clone data one fails to find any significant clone when using any reasonable FDR cut-off. This is a consequence of the fact that most multiple testing corrections are rather conservative when strong positive dependencies are present in the data (Benjamini and Yekutieli, 2001). However, the region data are less dependent by construction and we observe from Table 3 that, despite the small sample size of the MSI+ group, we find significant regions when using an FDR cut-off of 0.05. The regions largely overlap with those mentioned by Douglas et al. (2004), but statistical confidence is now provided. An exception is the region on chromosome 13, which is not mentioned by the authors. They do mention the 17p chromosomal arm, which in our analysis corresponds to an uncorrected *p*-value of 0.093 and an FDR-value of 0.20.

A positive side-effect of the reduction of the number of items to be tested is the saving in computing time, because this is approximately linear with the number of items. Here, computing time decreased from 72 sec. to 1.2 sec. using a PC with 1.73 Ghz processor and 1 Gb internal memory; for larger group sizes, computing time may reduce from a couple of hours to minutes. Also for oligonucleotide data, which contain many more clones, the ratio (#clones)/(#regions) is even larger and so is the saving in computing time. The major advantage, however, is the far less conservative FDR-control when reducing the clone data to regions, which results in more detection power and thus more statistically confident findings.

**Figure 2.**
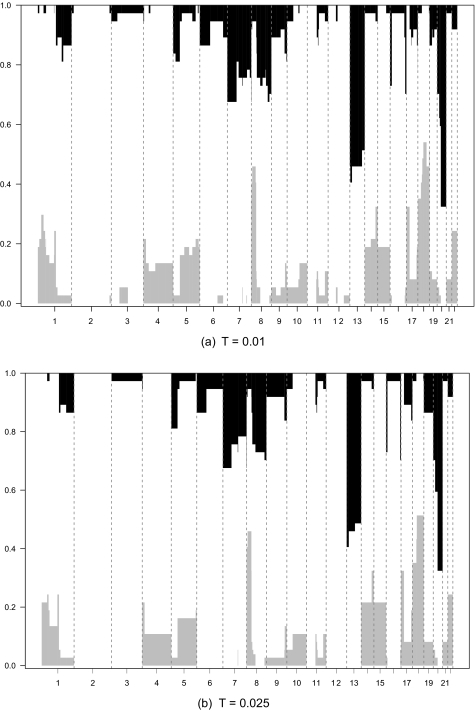
Region-wise frequency plots for 37 colorectal tumor samples (Douglas et al. 2004). Regions were created using settings *T* = 0.01 (**a**) and *T* = 0.025 (**b**). Left-axis displays the loss-proportion; this scale should be reversed (‘1-’) to obtain the gain-proportion.

For a one-group approach, CGHregions may be of use to find focal regions that contain (many) more aberrations than the neighboring regions. Figure 2 shows the aberration frequency plots for the regions found using *T* = 0.01 and *T* = 0.025. While *T* = 0.01 results in approximately twice as many regions as for *T* = 0.025, no large differences are observed. A possible exception is chromosome 18 where the results for *T* = 0.01 imply a somewhat smaller focal loss-region than for *T* = 0.025. Note that both settings result in small regions at the far ends of chromosome 16 which contain more gains (~ 25%) than their neighbors.

The positive effect of using regions rather than individual clones was also observed for other data. In Smeets et al. (2006), we studied chromosomal aberrations in 24 head-and-neck squamous cell carcinomas (HNSCC), 12 of which were positive for human papillomavirus type 16 (HPV) and 12 were HPV-negative. We used a preliminary version of CGHregions to define regions and performed two-group testing for 12 HPV-positive versus 12 HPV-negative HNSCCs. In that study, at least four significant (FDR ≤ 0.1) regions were determined (see Table 2 in Smeets et al. 2006). Repeating the analysis on 4699 individual clones revealed one clone corresponding to unadjusted *p*-value equal to 0.00037 and FDR = 0.108. While this may still be significant, the clone with the one-but-smallest *p*-value, 0.0019, corresponds to FDR = 0.704. Hence, only one clone would be found using the clone-wise data, while four highly relevant clone regions were discovered.

## Discussion

A new algorithm was introduced to reduce the dimension of array CGH data while assuring the loss of information is marginal. The high resolution of the experimental platform implies that one wants to be able to find small genomic regions of interest. The ability to do so is not lost with our algorithm: small regions are created in genomic neighborhoods with many transitions at different nearby locations, and large regions are formed in genomic neighborhoods with very few transitions for the vast majority of the samples (see Figure 1). It was shown that using the regions resulting from CGHregions instead of separate clones may lead to statistically rigorous findings, even for relatively small sample sizes.

Currently, many calling methods do not automatically detect amplifications. Some guidelines for automatic detection are provided by Fridlyand et al. (2004). When amplifications are present in some profiles one has to consider the discrete level assigned to these amplifications. We simply set this to ‘2,’ hence twice the level of a gain. The algorithm itself remains unchanged for such four-class data.

In this paper, we performed hypothesis testing on the clones and the regions using the aberration calls rather than the continuous (segmented) log2-ratios. The reason for this is simple: interpretation. In the first case, one can conclude that the aberration levels differ when rejecting the null hypothesis, which has a clear interpretation. In the second case, since one has not translated the log2-ratios to copy number levels, one can only conclude that the mean log2-ratios differ between the groups, which lacks a clear interpretation.

We illustrated the potential benefit of the reduction algorithm in the context of statistical testing. More types of downstream analysis could benefit from the reduction. Integration with gene expression data may become easier, for example by focusing on (usually relatively) few highly active DNA regions. CGHregions is also advantageous for cluster analysis of the called array CGH data (van Wieringen et al. 2006b). The use of regions, rather than clones, as input of the cluster method introduces a natural and data-driven weighting in the clustering. Long ‘dull’ chromosomal areas with normal DNA copy number and small amplifications are weighted equally. One thus clusters on the relevant features of the data, without letting dull areas dominate the cluster output. We performed simulations which show that the use of regions improves the performance of clustering methods. Moreover, the number of regions, being only a small percentage of the number of clones, simplifies a manual screening for regions with differences in the copy number frequency tables between clusters.

Construction of regions from individual clone data in a multi-sample context has been addressed before by Diskin et al. (2006) and Rouveirol et al. (2006). However, the main objective of these studies is different from ours. Their main aim is to detect (preferably small) chromosomal regions with relatively many aberrations, after which a list of potentially interesting genes is generated by locating the ones in or near those regions. Rouveirol et al. (2006) mention the potential dimension reduction effect of their method for further downstream analysis, but no control of information loss is provided with their method. While we showed that our method can be useful to find focal regions (Figure 2), this was not formalized here; instead, we emphasize dimension reduction with control of information loss.

In summary, we demonstrated that, with almost no information loss, CGHregions reduces array CGH clone data to region data, which leads to much more powerful downstream data analysis of array CGH data.

## Figures and Tables

**Table 1.** Signatures and distances (d) to previous clone for 18 clones.

**Chr.**	**Clone**	**S1**	**S2**	**S3**	**S4**	**S5**	**S6**	**S7**	**S8**	**S9**	**S10**	**d**
1	1	1	0	0	1	1	0	1	0	1	1	10
1	2	1	0	0	1	1	0	1	0	1	1	0
1	3	1	0	0	1	1	0	1	0	1	1	0
2	4	−1	0	0	−1	−1	−1	−1	0	1	0	10
2	5	−1	0	0	−1	−1	−1	−1	0	1	0	0
2	6	−1	0	0	−1	−1	−1	−1	0	1	0	0
2	7	1	1	0	1	1	1	0	1	1	1	12
2	8	−1	0	0	−1	−1	−1	−1	0	1	0	12
2	9	−1	0	0	−1	−1	−1	−1	0	1	0	0
2	10	−1	0	0	0	0	−1	−1	−1	1	2	4
2	11	−1	0	0	0	0	−1	−1	−1	1	1	0
2	12	−1	0	0	0	0	−1	−1	−1	1	1	0
2	13	0	0	0	0	0	0	−1	−1	1	1	2
2	14	0	0	0	0	0	0	−1	−1	0	1	1
2	15	0	0	0	0	0	0	−1	−1	0	1	0
2	16	0	0	0	0	0	0	−1	−1	0	1	0
2	17	1	0	1	1	1	0	1	0	0	0	8
2	18	1	0	1	1	1	0	1	0	0	0	0

**Table 2.** Regions created using *c* = 2 and their medoid signatures.

**Chr.**	**Region**	**S1**	**S2**	**S3**	**S4**	**S5**	**S6**	**S7**	**S8**	**S9**	**S10**
1	1–3	1	0	0	1	1	0	1	0	1	1
2	(4,5,6,8,9)	−1	0	0	−1	−1	−1	−1	0	1	0
2	10–12	−1	0	0	0	0	−1	−1	−1	1	1
2	13–16	0	0	0	0	0	0	−1	−1	0	1
2	17–18	1	0	1	1	1	0	1	0	0	0

**Table 3.** Regions significantly different for MSI+ and CIN+ colorectal cancers.

**Chromosome**	**BP Position Start**	**BP Position end**	**Number of Clones**	***p*-value**	**FDR-value**
8	7938099	32678693	32	0.00166	0.01372
20	30814489	63589868	50	0.00464	0.03652
18	32413398	72886818	54	0.00618	0.03652
18	73991368	77615559	8	0.00618	0.03652
8	731200	6933218	14	0.00753	0.03652
8	34108046	35026137	2	0.01677	0.04491
18	225168	25700568	32	0.01961	0.04593
18	27315721	29970100	4	0.02259	0.04593
13	19104448	32907695	16	0.02264	0.04593
